# Percutaneous treatment of a aorto-caval fistula in a old high risk patient

**DOI:** 10.1186/1471-2482-12-S1-S32

**Published:** 2012-11-15

**Authors:** Antonio Rapacciuolo, Maria Carmen De Angelis, Elisa di Pietro, Roberto Puglia, Ettore Di Tommaso, Danilo Ruggiero, Bruno Amato, Gabriele Iannelli

**Affiliations:** 1 Department of Clinical Medicine, Cardiovascular and Immunological Sciences, Federico II University of Naples, Italy; 2 Department of General, Geriatric, Oncologic Surgery and Advanced Technologies, University “Federico II” of Naples, Italy

## Abstract

**Background:**

To remark the feasibility of endovascular treatment of an aorto-caval fistula in a old high risk patient with “hostile” abdomen for previous surgeries.

**Methods:**

In September 2009 a 81-years-old patient was admitted in emergency at our department because of abdominal pain and massive oedema of the lower extremities associated to dyspnoea (New York Heart Association (NYHA) functional class III). A CT scan showed an aorto-caval fistula involving the abdominal aorta below the renal arteries. This abnormal communication was likely due to the previous abdominal surgeries, was complicated by occlusion of the inferior vena cava at the diaphragm and was responsible for the massive oedema of the lower extremities. Because of unstable conditions and hostile abdomen the patient was considered unfit for conventional surgery and an endovascular approach was planned. After unsuccessful attempt by positioning of an Amplatzer vascular ring into the fistula, a Medtronic covered stent-grafts were implanted from the renal arteries to the both common iliac arteries. The patient had an impressive improvement characterized by a 18 Kg weight loss and a complete restoration of the functional capacity (from NYHA class III to NYHA class I) associated to a complete resolution of the lower extremities oedema as confirmed at the a month-CT-scan.

**Conclusion:**

Endovascular surgery of aorto-caval fistula represents a good option in alternative to conventional surgery mostly in old high risk patient.

## Methods

In the year 2002 a 74 years-old man was diagnosed with retroperitoneal fibrosis which had caused bilateral ureteral obstruction. The right kidney function was successfully restored by stent placement.

In the year 2003 the patient was subjected to left nephrostomy to restore kidney function but the treatment was complicated by capsular haematoma that was surgically removed. Thereafter the left kidney was not functioning anymore.

In October 2008 the patient was admitted to the emergency room because of small bowel sub-obstruction and underwent a surgical operation in order to remove the fibrotic adherences causing the symptoms. Despite the previous diagnosis, he had not been specifically treated for the retroperitoneal fibrosis.

In September 2009 the patient arrived at our observation for the first time. He was admitted in the emergency room because of abdominal pain and massive oedema of the lower extremities associated to dyspnoea.

On arrival at our Department, the patient related dyspnoea, New York Heart Association (NYHA) functional class III. He was awake and conversant. The pulse was 88 beats per minute and the blood pressure 150/80. The skin was cool and dry. On physical examination, his oxygen saturation was 96 % while he was breathing room air. He had massive oedema of both lower extremities.

**Figure 1:**
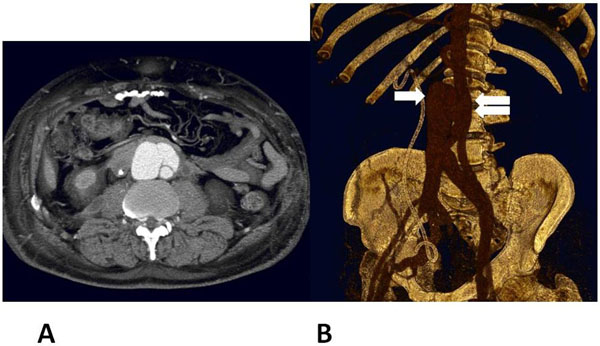
**A** Retroperitoneal fibrosis demonstrated at CT scan **B** Aorto-caval fistula originating from the abdominal aorta below the renal arteries (double arrow). The inferior vena cava (arrow) was completely occluded few centimetres above the communication with the aorta.

A CT scan was performed that evidenced retroperitoneal fibrosis (figure 1A) as well as an aorto-caval fistula involving the abdominal aorta below the renal arteries (figure 1B). This abnormal communication was likely due to the previous abdominal surgeries which had caused a hyatrogenic fistulous aorto-caval shunt. The inferior vena cava was completely occluded few centimetres above the communication with the aorta (figure 1B). This preserved the patient from massive pulmonary hyperafflux and was responsible for the massive oedema of the lower extremities.

The majority of Aorto-Caval Fistulas occur spontaneously, with most resulting from the rupture of an existing AAA into the vena cava or as result from iatrogenic injuries occurring during peripheral angiography, surgery or other potential etiologies [1].

Symptomatic ACFs have traditionally been repaired using open surgical techniques. These procedures can be technically challenging, with significant intraoperative blood loss and high operative morbidity and mortality rates approaching 30% [2].

The hostile abdomen with age and heart failure are a well recognized high-risk criteria for conventional surgery leading to a minimally invasive approach.

Since the patient had a surgically “hostile abdomen” due to retroperitoneal fibrosis, we decided to percutaneously treat the aorto-caval communication [3]. We selectively cannulated the aorto-caval fistula originating in the anterior wall of the aorta with a 7 FR guiding catheter and implanted an AMPLATZER® Vascular Plug II (AGA Medical Corporation), which is a unique multi-segmented, multi-layered design nitinol plug that significantly reduces occlusion time for transcatheter embolization procedures. The three adjustable lobes of the AMPLATZER Vascular Plug II are designed for enhanced conformability to vessel landing zones. We obtained a good angiographic result with almost complete abolishment of the shunt. In 3 days the patient had a 9 Kg weight loss associated to a significant reduction of the lower extremities oedema and of the dyspnoea.

**Figure 2:**
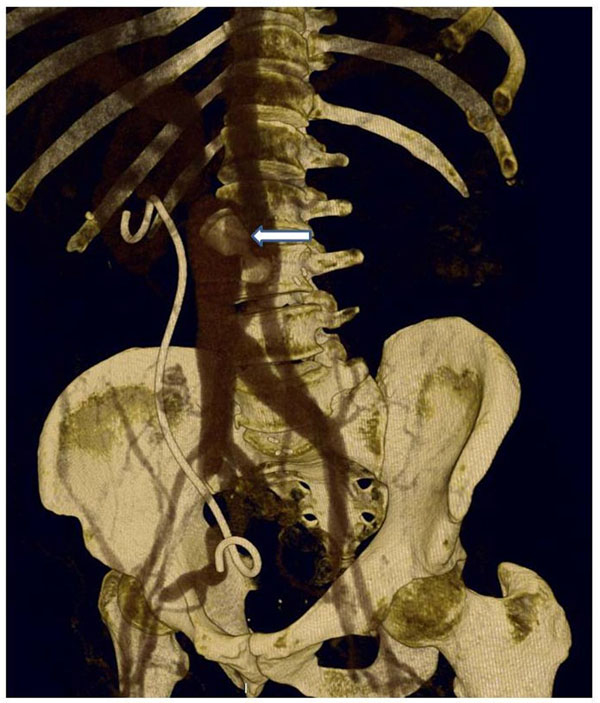
The CT scan at 1 month follow-up after vascular plug implantation showed recurrence of the aorto-caval communication. The Amplatzer vascular plug, still in correct position, was not able to halt the fistulous communication.

One month later the patient was regaining weight and there was a worsening of the symptoms. A new CT scan was performed which showed recurrence of the aorto-caval communication which was likely due to an additional fistula that was not closed with the initial procedure. The CT scan also showed the Amplatzer vascular plug still in correct position (fig. 2).

We therefore performed a new aortogram confirming the recurrence of the aorto-caval communication with the major shunt originating in the posterolateral left wall of the aorta. In order to obtain a complete occlusion of the aorto-caval communication, we decided to implant an aortic prosthesis Talent (Medtronic). The TalentTM Abdominal Stent Graft is designed to treat abdominal aortic aneurysms using a minimally invasive procedure called endovascular stent grafting. It is used to reinforce the weakened wall of the vessel to prevent the aneurysm from rupturing. The Talent Stent Graft is a woven polyester tube supported by a tubular metal web that expands to a pre-established diameter when placed in the artery [4,5].

**Figure 3:**
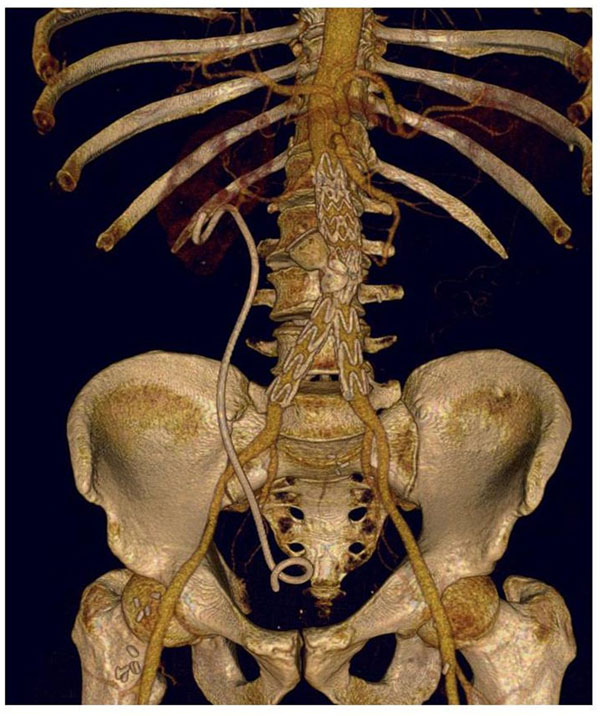
The CT scan 1 month after aortic endoprosthesis implantation showed a complete abolishment of the shunt with no opacization of the inferior vena cava from the abdominal aorta . The Amplatzer vascular plug was excluded from the aortic blood flow after the endoprosthesis implantation.

We first implanted an aortic stent graft that covered the abdominal aorta starting below the renal arteries and landing right before the aortic bifurcation. However, a residual shunt was still present. This residual fistulous communication was completely abolished by implanting an additional Talent device which would cover the abdominal aorta and both the iliac arteries. The patient had an impressive clinical course characterized by a 18 Kg weight loss and a complete restoration of the functional capacity (from NYHA class III to NYHA class I) associated to a complete resolution of the lower extremities oedema. The patient also initiated a specific therapy for retroperitoneal fibrosis with steroids and tamoxifen. The CT scan performed one month later showed a complete abolishment of the shunt with no opacization of the inferior vena cava from the abdominal aorta (Figure 3). The Amplatzer vascular plug was excluded from the blood flow after the endoprosthesis implantation (Figure 3).

## Conclusion

*Idiopathic* retroperitoneal fibrosis (IRPF) is a syndrome of unknown cause that typically presents with constitutional symptoms and back or abdominal pain. Ureteral obstruction is present in as many as 80% to 100% of reported cases. However, intestinal or bilary-pancreatic [6-8] or magnetic resonance imaging often shows periaortic soft tissue accumulation that encases the distal aorta below the renal arteries, as well as nearby structures [9-13].

The initial approach often requires surgical, urological, or medical intervention to address anatomic complications. For instance, ureteral obstruction usually respond to stent placement, bowel obstruction requires evaluation, observation and even diversion. However, when these complications have been addressed, medical treatment can be devised to treat the acute inflammation and suppress what appears to be a more chronic immunologic process with subsequent fibrosis.

In the present case, the previous surgical interventions had caused a hyatrogenic aorto-caval communication in a patient that had developed a very hostile abdomen to be treated with an additional surgical approach. Since the fistulous shunt was causing very important and life threatening symptoms, we decided to percutaneously intervene before starting an appropriate medical therapy. In fact, any kind of intervention for IRPF does not reduce long term prognosis if not associated to specific pharmacological therapy.

We first decided to close the abnormal communication by using a less invasive approach implanting an endovascular plug usually dedicated to vessel embolization. Previous experience have been reported treating aorto-caval fistulas with vascular plug implantation. However, the few cases reported in the literature refer to either fistulous communication resulting from a surgical intervention for aortic aneurysm or congenital aorto-caval fistula [3,14,15]. The present case was complicated from the presence of IRPF which would probably have caused multiple and complicated abnormal communication.

In fact, although the result was initially encouraging, the unfortunate recurrence of an important shunt aggravating the symptoms led us to consider an aortic endoprosthesis to definitely treat the inferior vena cava volume overload [16]. Aortic endoprosthesis have been successfully used to close aorto-caval communication in patients with either ruptured or dissected aortic aneurysm or penetrating abdominal trauma [2,17-21].

Aortic endoprosthesis implantation in the present case successfully treated the abnormal shunt resolving both lower extremities oedema and dyspnoea.

This is the first demonstration that such a percutaneous approach can be successfully used to treat a very complicated case of aorto-caval fistula with a device that is commonly used to treat aortic aneurysms.

## Competing interests

The authors declare that they have no competing interests.
